# Longitudinal changes in physical capacity from adolescence to middle age in men and women

**DOI:** 10.1038/s41598-018-33141-3

**Published:** 2018-10-03

**Authors:** M. Westerståhl, E. Jansson, M. Barnekow-Bergkvist, U. Aasa

**Affiliations:** 1 Division of Clinical Physiology, Department of Laboratory Medicine, Karolinska institutet, Stockholm, Sweden; 2 Department of Clinical Physiology, Karolinska University Hospital, Stockholm, Sweden; 3 Department of Epidemiology and Public Health, University of Umeå, Umeå, Sweden; 4 Department of Community Medicine and Rehabilitation, Umeå University, Umeå, Sweden

## Abstract

The aim was to investigate how physical capacity changes from adolescence through early adulthood to middle age with focus on early aging. The aim was also to study if physical capacity in middle age could be predicted by factors in adolescence or early adulthood. A cohort of men and women in Sweden (SPAF-1958, n = 425) have been followed for 36 years, at 16, 34, and 52 years of age. The study includes, among other variables, objective measures of physical capacity. At age 52, 50% of the original cohort participated in exercise testing. Physical capacity increased from 16 to 34 years. From 34 to 52 years, physical capacity decreased in both genders by 15–20% in all but one test. Physical capacity at 16 and 34 years of age were better predictors of physical capacity at age 52 than body dimensions, school grades and life style factors. In conclusion, present data confirm earlier cross-sectional studies regarding the decrease in aerobic capacity and muscular strength during the early ageing period in both genders. The study has also generated novel data that show a smaller decline in muscular endurance than previously reported. Finally, physical capacity is fairly stable from adolescence to middle age.

## Introduction

The physical capacity of an individual changes over their life, from adolescence through early adulthood and into middle age^1^. However, the longitudinal studies that show these changes in physical capacity are small or short-term studies^2–10^, and there is little confirmation based on long-term longitudinal studies with more than one time point of follow up.

It is important to maintain sufficient physical capacity into older ages in order to maintain one’s independence throughout life^11^. Maintaining one’s physical capacity is most likely also important for reducing morbidity and premature mortality^12–14^. Therefore, it is important to identify risk factors early in life for low physical capacity later in life in order to offer interventions to prevent an unhealthy decline in physical capacity.

In the longitudinal Swedish Physical Activity and Fitness cohort born in 1958 (SPAF-1958)^15^, our group has followed the same participants for 36 years in three waves of data collection (at 16, 34, and 52 years of age). The study includes, among other variables, measures of physical capacity, and the participants have answered questionnaires about their lifestyle. This offers the unique possibility to study longitudinal changes in physical capacity over a lifetime. The study also offers the possibility to study how characteristics in adolescence are associated with physical capacity in middle age. Three questions were focused upon in the present paper: (1) How does physical capacity change from adolescence to early adulthood and into middle age, with special focus on the changes in the early ageing period? (2) Are the changes in physical capacity similar for men and women? (3) Is physical capacity in middle age predicted by physical capacity, lifestyle, and/or educational background in adolescence or early adulthood?

## Methods

The SPAF-1958 study has a prospective, longitudinal design in which a systematically selected cohort of men and women in Sweden has been studied for 36 years. At baseline, the participants were selected from six geographic areas in Sweden^16,17^. The cohort was representative of Swedish 16-year-old pupils in their first year in upper secondary school in 1974^16^. The group was followed up in 1992^17–19^ and in 2010^15^. The study protocol was in accordance with the Helsinki Declaration and received ethical approval from the University of Umeå Human Research Ethics Committee, Dnr 09-082M. Participation was voluntary, and all participants signed an informed consent form.

### Participants

At 16 years of age, 220 boys and 205 girls were included in the study. At 34 and 52 years of age, the participants who lived in and around the six geographic areas were invited to come to the scheduled physical testing. At 34 years of age, 65% of the participants (men n = 157, women n = 121) were tested. At 52 years of age, 387 (90%) of the participants still lived in the areas and were invited for testing. Of these, 56% were tested (50% of the total cohort, total n = 213, men n = 114, women n = 99). Of these 213 participants, 83% had been tested at 34 years of age. The representativeness of these participants has been described previously^15^.

### Measurements

#### Anthropometry

Height and weight were measured at 16, 34, and 52 years of age without shoes and in light sports clothing. Body mass index (BMI, kg·m^−2^) and height^2^ (m^2^) were calculated. Normal weight was defined as a BMI up to 25 kg·m^−2^.

#### Aerobic capacity

At 16 years of age, the boys and girls performed a nine-minute run test in which the distance covered in nine minutes was measured in meters^20^. A subgroup of boys and girls also performed the submaximal exercise test on a cycle ergometer at 16 years of age. A regression equation for the conversion of the distance covered in the nine-minute run into relative aerobic capacity (ml·kg^−1^·min^−1^) was created from this subgroup and applied to the whole study group^21^. Also, the absolute aerobic capacity of the whole study group at the age of 16 was calculated (L·min^−1^). At 34 and 52 years of age, a submaximal exercise test on a cycle ergometer (Monark 828E, Monark Exercise AB, Sweden) was used to estimate maximal aerobic capacity (VO_2 max_). Absolute aerobic capacity (L·min^−1^) was estimated using an Åstrand nomogram and adjusted for age^22^. The relative aerobic capacity was calculated (ml·kg^−1^·min^−1^).

#### Muscular strength and muscular endurance

At 16, 34, and 52 years of age, muscular static strength was measured by the two-hand lift test and by the hand grip test. The two-hand lift test measures leg and back strength and was performed using a calibrated dynamometer^23^. Strength was expressed in Newtons (N) and was recorded as the better of two trials^17,24^. The hand grip test was performed in a standing position using a dynamometer. Different dynamometers were used at baseline and at the first follow up (Vigorimeter, Martin GmbH & Co KG, Gebrüder; kg·cm^–2^) and at the second follow up (Jamar dynamometer, Patterson Medical, Warrenville, Illinois, USA; kg), and therefore the longitudinal change in maximal static strength in the finger flexion muscles could not be analysed. The combined dynamic strength and endurance in arm and chest muscles was measured by the bench press test at 16, 34, and 52 years of age. The number of lifts to straight arms at a rate of 25 per minute was counted in one trial^24,25^. Different weights were used for men (20 kg) and women (12 kg) because the weights lifted were adjusted to an assumed gender difference. At 16 years of age, the dynamic endurance of the abdominal muscles was measured by a sit-up test^25^ and at 34 and 52 years of age by a curl-up test^17,24^. In both tests the participants’ hands were held behind the neck, and in the sit-up test they were asked to sit up to an upright position, while in the curl-up test the participants were to curl up above a stipulated mark. The number of sit-ups or curl-ups at a rate of 25 per minute was counted in one trial. At 34 and 52 years of age, static endurance of the back muscles was measured by the Biering–Sörensen back extension test (back endurance). The time until exhaustion was expressed in seconds in one trial^17,24^.

#### Lifestyle and educational background

The following questions were included in a questionnaire and used to evaluate the correlations with physical capacity at 52 years of age. Questions at 16 years of age were: (1) Do you participate in leisure time sports activities? (yes/no) (2) Are you a member of a sports club? (yes/no) (3) How often do you feel satisfied with your performance in physical education lessons? (5-point scale from “almost never” to “often”). Information about mean school grades at age 15 years was collected at 16 years of age. The grades were placed on a 5-point scale, where 5 was the highest and 1 was the lowest.

Questions at 34 years of age were: (1) Do you perform any physical activity during leisure time, including, for example, walks and gardening? (yes/no). (2) Do you walk or bike to work? If so, how many times and how many minutes per week do you do so? The number of minutes per week in active transportation to work was calculated by multiplying the duration by the frequency. (3) How do you perceive your physical activity compared to people your age? (5-point scale from “much lower” to “much higher”). (4) Do you smoke? (yes/no). (5) How do you perceive your aerobic capacity compared to other people your age? (5-point scale from “much lower” to “much better).

### Statistical methods

IBM SPSS Statistics for Windows version 24 (IBM Corp, Armonk, NY) was used for the statistical analyses. The outcome variables of bench press, curl-up, and back endurance were not assumed to be normally distributed^26^ according to their skewness >±1. Therefore, these variables were square root transformed to obtain normality in the analyses. For normally distributed variables, the mean and standard deviation are presented. For variables with skewed or limited distribution, the median and minimal-maximal values are presented (Table 1).

**Table 1:** Descriptive statistics for the anthropometrics and the physical capacity test at 16, 34 and 52 years of age.

		Men						Women					
		16 years		34 years		52 years		16 years		34 years		52 years	
		Mean (SD) or Median (25^th^–75^th^)	N	Mean (SD) or Median (25^th^–75^th^)	N	Mean (SD) or Median (25^th^–75^th^)	N	Mean (SD) or Median (25^th^–75^th^)	N	Mean (SD) or Median (25^th^–75^th^)	N	Mean (SD) or Median (25^th^–75^th^)	N
Anthropo-metrics	Weight (kg)	62.7 (9.1)	222	79.7 (10.2)	157	86.7 (13.4)	133	57.0 (7.8)	205	67.2 (12.0)	212	72.1 (13.8)	98
	Height (m)	1.76 (0.7)	222	1.80 (0.7)	157	1.80 (0.7)	112	1.66 (0.6)	205	1.68 (0.6)	212	1.68 (0.6)	98
	BMI (kg · m^−2^)	20.3 (2.4)	222	24.5 (2.7)	157	26.7 (3.5)	112	20.5 (2.4)	205	23.7 (3.7)	212	25.6 (4.3)	98
Aerobic capacity	Absolute VO_2 max_ (L · min^−1^)	2.5 (0.4)	213	3.3 (0.7)	157	2.8 (0.7)	100	2.0 (0.3)	196	2.7 (0.6)	212	2.4 (0.6)	88
	Relative VO_2 max_ (ml · kg-1 · min^−1^)	40 (5)^‡^	213	42 (9)	157	32 (8)	100	35 (5)^‡^	196	40 (10)	212	34 (10)	88
	Nine minute run (m)	2080 (296)	215	N/A		N/A		1627 (206)	196	N/A		N/A	
Muscular strength	Two-hand lift (N)	124 (23)	212	146 (22)	154	124 (26)	106	82 (17)	202	86 (16)	118	71 (19)	95
	Hand grip (kg · cm^−2^ at 16 and 34 years or kg at 52 years)^ϕ^	1.0 (0.3)	215	1.1 (0.2)	157	55 (9)	111	1.0 (0.2)	202	1.0 (0.2)	121	34 (6)	95
Muscular endurance	Bench press (number)	39 (30–50)	213	52 (40–70)	157	43 (35–56)	105	32 (26–40)	210	40 (30–53)	118	36 (26–48)	93
	Curl-up (number)	N/A		37 (28–56)	157	36 (24–59)	108	N/A		25 (16–33)	119	26 (17–38)	94
	Sit-up (number)	42 (30–60)	212	N/A		N/A		21 (16–30)	210	N/A		N/A	
	Back endurance (s)	N/A		142 (114–170)	157	101 (63–132)	107	N/A		149 (107–193)	120	106 (65–157)	92

SD = standard deviation, 25^th^–75^th^ = percentiles, N/A = not applicable because the test was not performed, ^ϕ^Different equipment were used so the absolute numbers are not comparable between the ages or between the genders. ^‡^The value at 16 years of age is estimated from the nine minute run test.

Student’s *t*-test was used to calculate the gender difference at each age.

#### Change in physical capacity with age

A linear mixed model analysis for models with uncorrelated terms was used to analyse the percentage changes in physical capacity. The percentage change in performance was set as the dependent variable and was calculated as ratios: the performance at 34 or 52 years of age was divided by the performance at 16 years of age, and the performance at 52 years of age was divided by the performance at 34 years of age. Gender was set as the independent variable and a significant gender effect was interpreted as a difference in percentage change between genders. The results from the nine-minute run test at 16 years of age and the question “Are you a member in a sports club (yes/no)” at 16 years of age differed systematically between study dropouts and study participants at 52 years of age^15^. Therefore, these two variables were entered as part of the analyses.

#### Drop out analysis

**Figure 1:**
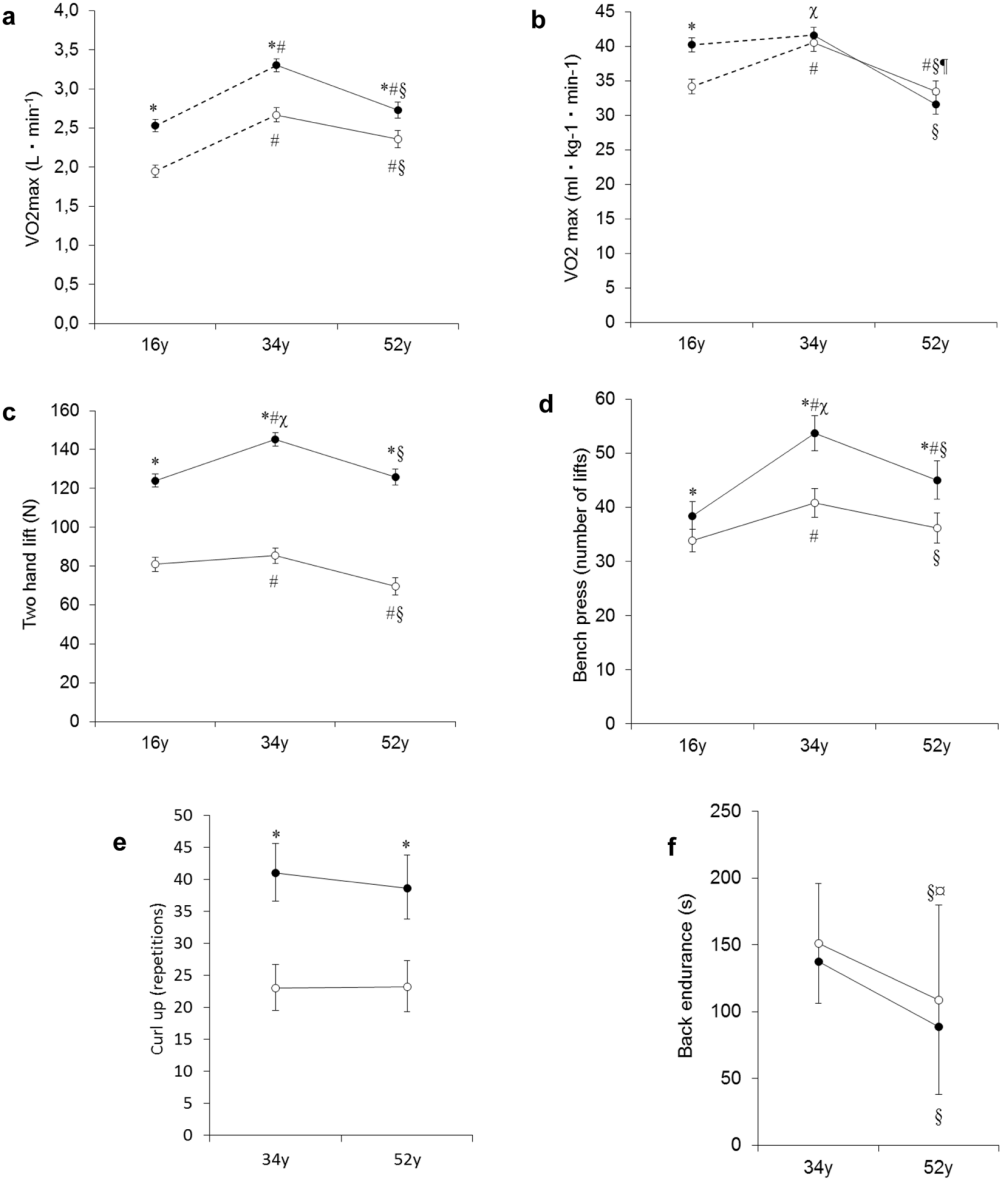
Physical capacity at 16, 34 and 52 years of age. y = years, N = Newton. Differences between groups are denoted as follows: *Significant gender difference, ^#^significant percentage change from 16 years (gender specific), ^§^significant percentage change from 34 years (gender specific), ^χ^significant gender difference in percentage change from 16 years, ^¤^Significant gender difference in percentage change from 34 years, (**a**) Absolute VO_2max._ All p-values < 0.001 with the exception for the percentage change in men from 16 to 52 years of age (p < 0.01). (**b**) Relative VO_2max_. All p-values < 0.001. (**c**) Two-hand lift. All p-values < 0.001 with the exception for the percentage change in women from 16 to 34 years of age (p < 0.01). (**d**) Bench press. All p-values < 0.001 with the exception for the percentage change in women in bench press from 34 to 52 years of age (p < 0.01). (**e**) Curl-up. All p-values < 0.001. (**f**) Back endurance. All p-values < 0.001 with the exception for the gender difference in percentage change (p < 0.05).

Linear mixed models analysis for models with correlated random effects were used to calculate adjusted mean values for the physical capacity tests by entering the variables that differed systematically between study dropouts and study participants at 52 years of age (mentioned above) in the analysis. The adjusted mean values are presented in Fig. 1. The adjustments lowered the mean values by on average two percent.

To further explore if the participants at 52 years of age were representative of the original study cohort, we explored the characteristics of a subgroup of the dropouts at 52 years of age. A total of 93 men and women completed a questionnaire at 52 years of age, but did not participate in the physical capacity testing. We found that the performance in most of the physical capacity tests at 52 years of age differed between leisure time physically active and inactive subjects at the age of 52. Therefore, three groups were defined: the test group, which included the subjects that participated in the physical capacity testing at 52 years of age; the dropout group A that completed a questionnaire at 52 years of age, but did not participate in the physical capacity testing; the dropout group B that included all participants at baseline that did not participate at the testing at 52 years of age (n = 212). In dropout group A there were more leisure time physically inactive subjects at 52 years of age than in the test group (39% compared to 25% physically inactive subjects as characterized by the questionnaire, see the question above). On the assumption that dropout group B included a similar proportion of inactive subjects as dropout group A and the fact that the test group and the dropout group B were of equal size, the whole baseline cohort could be estimated to include 32% inactive subjects at 52 years of age ((39% + 25%) · 0.5). The physical capacity at 52 years of age was higher among the subjects that were leisure time physically active for all tests except the muscular strength tests (two-hand lift and hand grip). Relative VO_2 max_ at 52 years of age was 29 ml·kg·min^−1^ in the physically inactive group and 34 ml·kg·min^−1^ in the active group. By using these VO_2 max_ values and the frequency of physically inactive and active subjects in the two groups, the VO_2 max_ for the whole baseline cohort was estimated to be 32 ml·kg·min^−1^ (32% · 29 ml·kg·min^−1^ + 68% · 34 ml·kg·min^−1^), i.e. two percent lower than the mean values presented for the test group. In a similar way, we calculated that the performance in both the bench press and curl-up test were around three percent lower in the whole cohort than in the test group. The two-hand lift and hand grip performance did not differ between the whole cohort and the test group (data not shown). Based on these calculations, we assume that the mean values for the physical capacity in the present study represent the performance at 52 years of age of the baseline study cohort satisfactory.

#### Prediction of physical capacity in adulthood

To determine the predictive power of each physical capacity test at 16 and 34 years of age on the same physical capacity test at 52 years of age, the inter-age correlation (i.e. the tracking) was analysed using a linear regression analysis. The effect of body size in the analysis of inter-age correlation was minimized by adjusting the analyses for height^2^ (m^2^) at 52 years of age except for the variable of relative aerobic capacity, which is already related to body size. Adjustments were also made for gender in all analyses. Inter-age correlations were defined as low (r < 0.3), moderate (r = 0.3–0.6), or good (r > 0.6)^27^. The standardized beta coefficient is presented to enable an estimation of the magnitude of inter-age correlation from 16 or 34 years to 52 years of age (Table 2). To analyse the predictive value of BMI, lifestyle, and educational background at 16 or 34 years of age on physical capacity at 52 years of age, the physical capacity measure at 52 years of age was set as the dependent variable in a linear regression analysis. The predictors were the variables described previously in the section “Lifestyle and educational background” and the variable BMI. Each predictor was tested individually in the linear regression analysis after adjustment for gender and height^2^. To evaluate how much of the variance of the physical capacity at 52 years of age was explained by each independent variable, the partial correlations were squared and multiplied by 100 to yield a percentage value. The unstandardized beta of each predictive variable at 16 or 34 years of age on performance at 52 years of age is presented.

**Table 2:** Interage correlations and effect sizes for physical capacity between 16 and 52 years of age and between 34 and 52 years of age.

Variabel		16 and 52 years of age		34 and 52 years of age	
		Interage correlation	Effect size (Standardized Beta)	Interage correlation	Effect size (Standardized Beta)
Aerobiccapacity	Absolute VO_2 max_ (L · min^−1^)	0.28***^,‡^	0.34	0.46***	0.50
	Relative VO_2 max_ (mL · kg^−1^ · min^−1^)	0.22**^,‡^	0.24	0.53***	0.54
Muscularstrength	Two-hand lift (N)	0.30***	0.28	0.58***	0.68
	Hand grip (kg)	0.34***	0.19	0.41***	0.27
Muscularendurance	Bench press (number)	0.44***	0.44	0.66***	0.67
	Curl-up (number)	0.19*	0.21	0.55***	0.60
	Back endurance (s)	N/A	N/A	0.58***	0.57

*p < 0,05, **p < 0,01, ***p < 0,001, N/A = not applicable because the test was not performed at 16 years of age. ^‡^The value at 16 years of age is estimated from the nine minute run. Gender and height^2^ at 52 years of age are included in all analyses except for the analyses of aerobic capacity (see Methods, Statistical analysis).

Differences were regarded as statistically significant if p < 0.05.

## Results

The results from the anthropometric measurements and the physical capacity tests at 16, 34, and 52 years of age are presented in Table 1. The men had higher weight, height, and BMI than the women at all three measurement occasions, except for BMI at 16 years of age for which there was no difference (p = 0.2). At 16 years of age, 96% of the participants were normal weight, at 34 years of age 64% of the men and 74% of the women were normal weight, and at 52 years of age 29% of the men and 48% of the women were normal weight.

### Change in performance with age

#### Aerobic capacity

VO_2 max_ was estimated by a submaximal cycle ergometer test at 34 and 52 years of age. At 16 years of age VO_2 max_ was estimated from a nine-minute run test.

Absolute VO_2 max_ (L · min^−1^) (Fig. 1): From 16 to 34 years of age, absolute VO_2 max_ increased by a similar percentage in both genders (38% increase in women and 31% increase in men). From 34 to 52 years of age, absolute VO_2 max_ decreased by a similar percentage in both genders (12% decrease in women and 17% decrease in men). Absolute VO_2 max_ was lower in women than in men at all ages (23% lower at 16 years, 19% lower at 34 years, and 13% lower at 52 years).

Relative VO_2 max_ (mL · kg^−1^ · min^−1^) (Fig. 1): From 16 to 34 years of age, the percentage change in relative VO_2 max_ differed between the genders. The relative VO_2 max_ increased in women (19% increase) but not in men. From 34 to 52 years, relative VO_2 max_ decreased by a similar percentage in both genders (18% decrease in women and 24% decrease in men). Relative VO_2max_ was lower in women than in men at 16 years (15% lower), but not at 34 years or 52 years of age.

#### Muscular strength and muscular endurance

Two-hand lift (N) ***(***Fig. 1): The two-hand lift test was performed at 16, 34, and 52 years of age. From 16 to 34 years of age, the percentage increase in the performance in the two-hand lift test was smaller in women than in men (7% increase in women and 20% increase in men). From 34 to 52 years of age, the performance in the two-hand lift test decreased by a similar percentage in both genders (15% decrease in women and 14% decrease in men). The performance in the two-hand lift was lower in women than in in men at all ages (35% lower at 16 years, 41% lower at 34 years, and 45% lower at 52 years).

Hand grip (kg · cm^−2^): Different dynamometers were used at baseline and at follow ups. Also, different dynamometers were used for women and men at 16 and 34 years of age. Therefore, neither the longitudinal change nor the gender difference at 16 or 34 years of age in hand grip strength could be analysed. The hand grip strength was 38% lower in women than in men at 52 years of age.

Bench press (number) (Fig. 1): The bench press test was performed at 16, 34, and 52 years of age. From 16 to 34 years, the percentage increase in bench press performance was smaller in women than men (9% increase in women and 22% increase in men). From 34 to 52 years, the bench press performance decreased by a similar percentage in both genders (7% in both women and men). The bench press performance was lower in women than in men at all ages, although women and men lifted different weights (12% lower at 16 years, 24% lower at 34 years, and 20% lower at 52 years).

Curl-up (number) (Fig. 1): At 16 years of age, the sit-up test was performed, while at 34 and 52 years of age the curl-up test was performed. Therefore, the longitudinal change was only evaluated between 34 to 52 years of age. From 34 to 52 years of age, there was no change in curl-up performance in women or men. Women performed fewer curl-ups compared to men both at 34 years (30% fewer) and at 52 years of age (24% fewer). At 16 years of age, women performed significantly fewer sit-ups compared to men (28% fewer).

Back endurance (s) (Fig. 1): The back endurance test was performed at 34 and 52 years of age. From 34 to 52 years, the decrease in back endurance was smaller in women than men (15% decrease in women and 21% decrease in men). Back endurance was similar in women and men both at 34 years and at 52 years.

### Prediction of adult performance

#### Tracking of physical capacity

There was a low to moderate tracking of physical capacity (adjusted for gender and height^2^) from 16 to 52 years of age with inter-age correlations between 0.2 and 0.4 (Table 2). There was a moderate to good tracking from 34 to 52 years of age with inter-age correlations between 0.4 and 0.7. The standardized beta coefficients ranged from 0.2 to 0.4 for the tracking from 16 years of age and between 0.3 and 0.7 for the tracking from 34 years of age. A total of 5–19% of the variances in the physical capacity tests at 52 years of age were predicted by performance in the same test at 16 years of age, and 17–44% of the variances were predicted by the performance at 34 years of age.

#### Non-physical capacity factors

A total of 2–7% of the variances in the physical capacity tests at 52 years of age were predicted by variables at 16 years of age, and 2–10% were predicted by variables at 34 years of age (Table 3). In short, aerobic capacity at 52 years of age was positively predicted by measures of physical activity at both 16 and 34 years of age and negatively predicted by smoking at 34 years of age. Muscular endurance at 52 years of age was positively predicted by measures of physical activity and measures of physical capacity at both 16 and 34 years of age, by BMI at 16 years of age (positively), by BMI at 34 years of age (negatively), and by higher mean grades at 16 years of age (positively). Muscular strength was positively predicted by BMI at 16 years of age, but not by any of the measured factors at 34 years of age.

**Table 3:** Predictors at 16 or 34 years of age for the physical fitness at 52 years of age.

Physical capacity test at 52 years of age	Absolute VO_2_ (L · min^−1^)	Relative VO_2_ (ml · kg^−1^ · min^−1^)	Two-hand lift (kg)	Hand grip (kg)	Bench press (number)^#^	Curl-up (number)^#^	Back endurance (s)^#^
**Predictor at 16 years of age**							
LTPA (yes vs. no)	0.3L · min^−1^ higher VO_2_ (6%**)	3 mL · kg^−1^ · min^−1^ higher VO_2_ (2%*)	—	—	11 repetitions more benchpress (6%**)	—	
Satisfaction at PE (highest vs. Lowest, 5-grade scale)	0.8 L · min^−1^ higher VO_2_ (7%**)	11 mL · kg^−1^ · min^−1^ higher VO_2_ (5%**)	—	—	—	—	
Mean school grades (highest vs. lowest, 5-grade scale)	—	7 ml · kg^−1^ · min^−1^ higher VO_2_ (2%*)	—	—	13 repetitions more benchpress (2%*)	—	42 seconds longer back endurance (2%*)
BMI (per extra unit BMI, kg · m^−2^)	—	—	1.6 kg higher THL (2%*)	0.5 kg higher grip strength (3%*)	1 repetitions more benchpress (2%*)	—	—
**Predictor at 34 years of age**							
LTPA (yes vs. no)	0.3L · min^−1^ higher VO_2_ (6%**)	4 ml · kg^−1^ · min^−1^ higher VO_2_ (4%**)	—	—	7 repetitions more benchpress (2%*)	15 repetitions more curlups (5%**)	
Self-perceived PA compared to peers (much higher compared to much lower, 5-grade scale)	0.7 L · min^−1^ higher VO_2_ (5%**)	3 ml · min^−1^ higher VO_2_ (7%***)	—	—	5 repetitions more benchpress (4%**)	3 repetitions more curlups (5%**)	13 seconds longer back endurance (4%**)
Active transport to work (per extra 10 min · week^−1^)	0.1 L · min^−1^ higher VO_2_ (5%**)	2 ml · min^−1^ · kg^−1^ higher VO_2_ (7%***)	—	—	—	—	—
BMI (per extra unit BMI, kg · m^−2^)	—	0.4 ml · min^−1^ · kg^−1^ lower VO_2_ (9%***)	—	—	—	4 repetitions less curlups (8%***)	6 seconds shorter back endurance (6%**)
Self-percieved aerobic capacity compared to peers (highest vs. lowest)	—	—	—	—	—	5 repetitions more curlups (4%**)	19 seconds longer back endurance (7%**)
Smoking (no vs. yes)	0.3L · min^−1^ higher VO_2_ (3%*)	—	—	—	—	—	—

Each predictor was tested individually in a regression analysis after adjustment for gender and height^2^. The variance that each predictor explains is presented in brackets.

*p < 0,05, **p < 0,01, ***p < 0,001, [—] indicates that there was no significant association betweenthe variables. ^#^Analyses were performed on square-root transformed numbers, however, the numbers presented for the effect of predictor are derived from analyses of non-transformed numbers. y = years of age, THL = two-hand lift, PE = physical education, LTPA = Leisure-time physically active. Height^2^ at 52 years of age and gender were included as independent factors in all models (see Methods, Statistical analysis). The exception was relative VO_2 max_ where height^2^ was not included.

## Discussion

To our knowledge, this is the first study to objectively measure physical capacity in the same individuals at three time points, from adolescence to middle age, with a total follow up time of 36 years.

Three questions were focused upon in the present paper: (1) How does physical capacity change from adolescence to early adulthood and into middle age, with special focus on the changes in adulthood? (2) Are the changes in physical capacity similar for men and women? (3) Is physical capacity in middle age predicted by physical capacity, lifestyle, and/or educational background in adolescence or young adulthood?.

In summary, from 16 to 34 years of age, the performance increased in all tests. The percentage increase was larger in men than in women for muscular endurance and strength, similar in both genders for absolute VO_2 max_ (L · min^−1^), and larger in women than men for relative VO_2 max_ (mL · kg^−1^ · min^−1^). From 34 to 52 years of age, the performance decreased in all tests except for curl-ups. The percentage decrease was similar in men and women, except for the back endurance test, where the percentage decline was larger in men than in women. Physical capacity at the age of 52 years was predicted by physical capacity at the ages of 16 years (low to moderate tracking) and 34 years (moderate to high tracking). Physical capacity at the age of 52 was also predicted by non-physical capacity factors, but these had a lower predictive value than the physical capacity tests.

Three strategies were used to evaluate if the participants in the test group at the second follow up were representative of the original study cohort^28^. In the statistical analyses we adjusted the results from the physical capacity testing, for the known differences at 16 years of age between the dropouts and the test group. We also evaluated the representativity of the test group by using our knowledge about a subgroup of the dropouts (see methods). Lastly, we compared the characteristics of the participants with large national surveys to evaluate the representativity of our group^15^. We found that the participants were comparable in BMI, perceived health, smoking, physical activity level and aerobic capacity to other large national surveys^15^. In summary, we estimated that the participants at 52 years of age were reasonable representative of the original study cohort and also similar in many aspects to subjects in large national surveys.

We analysed the longitudinal changes in physical capacity and found a greater increase in muscular strength and muscular endurance from 16 to 34 years of age among men compared to women, and this has been described in detail in a previous publication from our group^17^. The results were similar to other longitudinal studies that included an assessment of muscular capacity in the adolescent years^3,5,6,29,30^. Such a gender difference in the development of muscular capacity from adolescence to early adulthood is most likely due to the higher age for the peak strength velocity in men than in women^3,31^. However, the increase in both genders in absolute aerobic capacity (L · min^−1^) from 16 to 34 years seems large considering that the aerobic capacity was measured at least two years after the peak velocity in aerobic capacity^32^. A systematic error between the measurements of aerobic capacity at 16 years and at 34 years of age cannot be excluded because the aerobic capacity was estimated by different methods at these two points of time.

We also analysed the longitudinal changes in physical capacity from 34 to 52 years of age, an early ageing period. This has, to the best of our knowledge, not previously been reported in a longitudinal setting. Our results confirm the findings from cross-sectional studies that show a 15–20% decline in aerobic capacity^8,33^ and muscular strength^34,35^ from 30 to 50 years of age. However, with regard to the change in muscular endurance from early adulthood to middle age, we could not confirm the findings from cross-sectional studies. Cross-sectional studies of the curl-up test indicate a more pronounced decrease in muscular endurance than was seen in the present study. The performance in the curl-up test did not change from 34 to 52 years of age in the present study, while the curl-up performance decreased by 30–60% in two cross-sectional studies that included similar age groups^36,37^. The numbers of curl-ups performed were generally lower in all age groups in the two previous studies compared to the participants in the current study. Also, with regard to the change in back endurance in adulthood, women had a smaller decrease in back endurance compared to men in the present study. This is in contrast to cross-sectional findings where women seemed to decrease more in back endurance in adulthood compared to men^38,39^. The reasons for the difference in the magnitude of the decline in muscular endurance in the early ageing period between the present longitudinal study and the cross-sectional studies are unknown but might partly be attributed to the differences in study cohorts, differences in time trends, or to systematic error in the testing. However, we cannot rule out that the longitudinal setting presents new insights into the change in muscular endurance in the early ageing period, showing a smaller decline in muscular endurance in the early ageing period than previously indicated.

The design of this study offers the possibility to study how characteristics in adolescence and adulthood are associated with physical capacity in middle age. There was in general a low to moderate predictive value of physical capacity in adolescence or early adulthood into middle age, with inter-age correlations of 0.2–0.3 between 16 and 52 years of age (36 years of follow up) and correlations of 0.4–0.6 between 34 and 52 years of age (18 years of follow up). Other studies with follow up times between 8 and 25 years have reported similar inter-age correlations of aerobic capacity^4–6,29,40–42^ and muscular capacity^5,41^ from adolescence to young adulthood as in the present study. Thus, the individual performance in physical capacity tests, in particular in muscular strength at 34 years of age, remained relatively stable over the next 18 years.

In the present study, physical capacity tests at 16 and 34 years of age were better predictors of physical capacity at the age of 52 years than body dimensions, school grades and life style factors. This was an expected finding because these non-physical capacity factors are measured subjectively and with less precision. We found that a total of 2–8% of the variance in the physical capacity at 52 years of age could be explained by non-physical capacity factors such as the self-reported measures of physical activity, mean school grades, BMI, and smoking at 16 or 34 years of age. Despite the low degree of variance that these factors explained, these factors might still be of importance for future health. For example, in the present study the aerobic capacity at 52 years of age was 3–4 mL · kg^−1^ · min^−1^ higher among those who were physically active at 16 or 34 years of age compared to those who were not physically active. Studies show that 1–1.5 MET (metabolic equivalent, approximately 3.5 mL · kg^−1^) higher aerobic capacity is associated with a 10–30% reduction in the incidence of adverse cardiovascular events^12^ and cardiovascular risk factor clustering^33^. Also, higher mean school grades and feeling satisfied with participation in physical education in adolescence predicted higher aerobic capacity at 52 years of age. These factors might be proxy measures for the cultural capital and the socio-economic status that are associated with being physically active^43,44^. Also, a high level of muscular strength is very likely to be important for health because muscular strength is associated with a lower risk of mortality and morbidity^45^. Apart from the moderately high tracking of muscular strength in the present study, only BMI at 16 years of age positively predicted muscular strength at 52 years of age. Only a few percent of the cohort were overweight at 16 years of age in 1974, and a higher BMI most likely reflected maturity and relative fat-free mass, both of which are positively associated with strength performance. Regarding the factors that could predict the muscular endurance at 52 years of age, BMI at 34 years of age was negatively associated with the curl-up and back endurance performance. At 34 years of age, 25–35% of the cohort were overweight with a high BMI most likely reflecting a higher relative fat mass, which is negatively associated with endurance performance^46^.

One of the strengths of this study is that, in comparison to previous longitudinal studies that measured physical capacity objectively, the original study cohort was a large group that represented all 16-year-old Swedish boys and girls in upper secondary school^15^. Another strength is that physical capacity was measured longitudinally and objectively at 16, 34, and 52 years of age, and that some of the tests were the same at all three testing occasions. Also, one of the researchers (EJ) was involved in all three data collections and one was involved in the two most recent data collections (MBB). A further strength of the study is that we took into consideration the small but systematic effect of the dropout in our analysis of the changes in performance with age. In longitudinal studies with long follow up periods, there are high risks for dropout and problems arise when the results are biased by differential dropout of participants. Therefore, we evaluated the representativity of the participants of the second follow up in three different ways as described above. One of the limitations of the study is that about 25% of the adolescents living in the areas at the time did not attend upper secondary school and thus are not represented in this cohort. Because educational level is positively associated with physical capacity, this means that even though the participants at 52 years of age were representative of the baseline cohort, the physical capacity might still be overestimated in our cohort compared to the average Swedish man and woman. However, the study cohort at the second follow up was one of the largest groups of 50-year-old men and women with objectively measured physical capacity in Sweden, and we estimate this cohort to be reflective of the average middle-aged man and woman in Sweden.

In conclusion, this longitudinal study from adolescence to middle age, with a total follow up time of 36 years, has confirmed earlier cross-sectional studies regarding the 15–20% decrease in the early ageing period in aerobic capacity and muscular strength. This study has also generated novel data that show a smaller decline than previously reported in muscular endurance, and that the decrease in physical capacity was similar in men and women. Physical capacity seems to be fairly stable from adolescence to middle age, and physical capacity in adolescence and early adulthood were better predictors of physical capacity in middle age than body dimensions, school grades and life style factors.
